# Antibiotic Bactericidal Activity Is Countered by Maintaining pH Homeostasis in *Mycobacterium smegmatis*

**DOI:** 10.1128/mSphere.00176-16

**Published:** 2016-08-24

**Authors:** I. L. Bartek, M. J. Reichlen, R. W. Honaker, R. L. Leistikow, E. T. Clambey, M. S. Scobey, A. B. Hinds, S. E. Born, C. R. Covey, M. J. Schurr, A. J. Lenaerts, M. I. Voskuil

**Affiliations:** a Department of Immunology and Microbiology, University of Colorado—Denver, School of Medicine, Aurora, Colorado, USA; b Department of Anesthesiology, University of Colorado—Denver, School of Medicine, Aurora, Colorado, USA; c Mycobacteria Research Laboratories, Department of Microbiology, Immunology and Pathology, Colorado State University, Fort Collins, Colorado, USA; Antimicrobial Development Specialists, LLC

**Keywords:** Antibiotics, bactericidal activity, mycobacteria, pH homeostasis

## Abstract

Antibiotics target specific biosynthetic processes essential for bacterial growth. It is intriguing that several commonalities connect the bactericidal activity of seemingly disparate antibiotics, such as the numerous conditions that confer broad-spectrum antibiotic tolerance. Whether antibiotics kill in a manner unique to their specific targets or by a universal mechanism is a critical and contested subject. Herein, we demonstrate that the bactericidal activity of diverse antibiotics against *Mycobacterium smegmatis* and four evolutionarily divergent bacterial pathogens was blocked by conditions that worked to maintain intracellular pH homeostasis. Single-cell pH analysis demonstrated that antibiotics increased the cytosolic pH of *M. smegmatis*, while conditions that promoted proton entry into the cytosol prevented intracellular alkalization and antibiotic killing. These findings led to a hypothesis that posits antibiotic lethality occurs when antibiotics obstruct ATP-consuming biosynthetic processes while metabolically driven proton efflux is sustained despite the loss of proton influx via ATP synthase. Consequently, without a concomitant reduction in respiratory proton efflux, cell death occurs due to intracellular alkalization. Our findings indicate the effects of antibiotics on pH homeostasis should be considered a potential mechanism contributing to antibiotic lethality.

**IMPORTANCE** Since the discovery of antibiotics, mortality due to bacterial infection has decreased dramatically. However, infections from difficult to treat bacteria such as *Mycobacterium tuberculosis* and multidrug-resistant pathogens have been on the rise. An understanding of the cascade of events that leads to cell death downstream of specific drug-target interactions is not well understood. We have discovered that killing by several classes of antibiotics was stopped by maintaining pH balance within the bacterial cell, consistent with a shared mechanism of antibiotic killing. Our findings suggest a mechanism of antibiotic killing that stems from the antibiotic’s ability to increase the pH within bacterial cells by disrupting proton entry without affecting proton pumping out of cells. Knowledge of the core mechanism necessary for antibiotic killing could have a significant impact on the development of new lethal antibiotics and for the treatment of recalcitrant and drug-resistant pathogens.

## Abstract (precis)

Since the discovery of antibiotics, mortality due to bacterial infection has decreased dramatically. However, infections from difficult to treat bacteria such as *Mycobacterium tuberculosis* and multidrug-resistant pathogens have been on the rise. An understanding of the cascade of events that leads to cell death downstream of specific drug-target interactions is not well understood. We have discovered that killing by several classes of antibiotics was stopped by maintaining pH balance within the bacterial cell, consistent with a shared mechanism of antibiotic killing. Our findings suggest a mechanism of antibiotic killing that stems from the antibiotic’s ability to increase the pH within bacterial cells by disrupting proton entry without affecting proton pumping out of cells. Knowledge of the core mechanism necessary for antibiotic killing could have a significant impact on the development of new lethal antibiotics and for the treatment of recalcitrant and drug-resistant pathogens.

## INTRODUCTION

Antibiotics target biosynthetic processes essential for cell growth. Inhibition of nucleic acid, protein, or cell wall synthesis leads to cessation of growth and often cell death. However, why some drugs kill while others are bacteriostatic is not well understood. Additionally, the specific downstream effects of the primary drug-target interaction that ultimately cause bacterial death have yet to be elucidated. Multiple commonalities exist between the bactericidal activities of seemingly disparate antibiotic targets. These include the existence of generalized persister populations that are simultaneously tolerant to multiple antibiotics as well as specific metabolic conditions that confer broad-spectrum antibiotic tolerance, particularly those that inhibit respiration (1 – 8). The existence of these commonalities has led to the hypothesis that antibiotics share a mechanism of bactericidal activity (3, 11).

Although the exact mechanism of antibiotic cell death is unclear, it appears that cellular metabolism plays a key role. Cellular processes involved in metabolism, growth, division, and maintaining energy balance are multifaceted and numerous. Cellular respiration in its simplest form can be described as a series of metabolic reactions used to break down nutrients in order to provide biochemical energy in the form of ATP. Electron carriers such as NADH feed electrons from catabolic reactions to electron transport systems, which translocate protons across the inner cytoplasmic membrane, forming a proton gradient. Energy from the proton gradient is harnessed via ATP synthase as protons flow back into the cell (10). Vast amounts of ATP are then hydrolyzed during biosynthetic processes such as nucleic acid, protein, and cell wall synthesis. It is well established that slowing respiration will result in general antibiotic tolerance (3 – 5, 11 – 13). More broadly, conditions that increase tolerance to antibiotics appear to have one commonality: a decreased capacity for proton translocation. Inhibition of cytochrome oxidase or other respiratory components will result in reduced electron flow through the electron transport system (ETS), directly reducing mechanisms for pH gradient generation (4, 5, 13 – 15). Metabolic manipulations may also affect proton translocation indirectly through actions on pathways that in turn decrease turnover of NADH or other mechanisms that donate electrons to the ETS (3, 6, 11).

We propose a model in which an imbalance in proton homeostasis, resulting in a decrease in intracellular proton concentration, is at least partially responsible for antibiotic-induced cell death. The majority of protons that reenter the cell do so through ATP synthase (10, 14), thus maintaining proton homeostasis. ATP synthesis only occurs if ATP is consumed by biosynthetic processes that recycle ADP. If major ATP-driven biosynthetic processes are inhibited by antibiotic activity, ATP turnover will decrease. A decrease in proton influx coupled with continued or increased proton efflux should cause an increase in intracellular pH (pH_IN_), which if substantial enough could result in bacterial membrane disruption.

We challenged Gram-positive, Gram-negative, and acid-fast bacteria with multiple classes of antibiotics to study the role of proton homeostasis in antibiotic-induced cell death. We demonstrated that antibiotic-induced death was abrogated by conditions that lowered pH_IN_ and was exacerbated under conditions in which pH_IN_ increased. Using flow cytometry for single-cell analysis of *Mycobacterium smegmatis*, pH_IN_ was observed to increase after antibiotic exposure but not in the presence of a protonophore, which decreased pH_IN_ and rescued antibiotic killing. The observed decrease in antibiotic killing resulting from manipulation of pH homeostasis did not appear to be a result of a general disruption of metabolic processes or decreased antibiotic activity against specific antibiotic targets.

## RESULTS

### **Protonophore activity and acidic extracellular pH prevented chlorpromazine lethality**.

**FIG 1:**
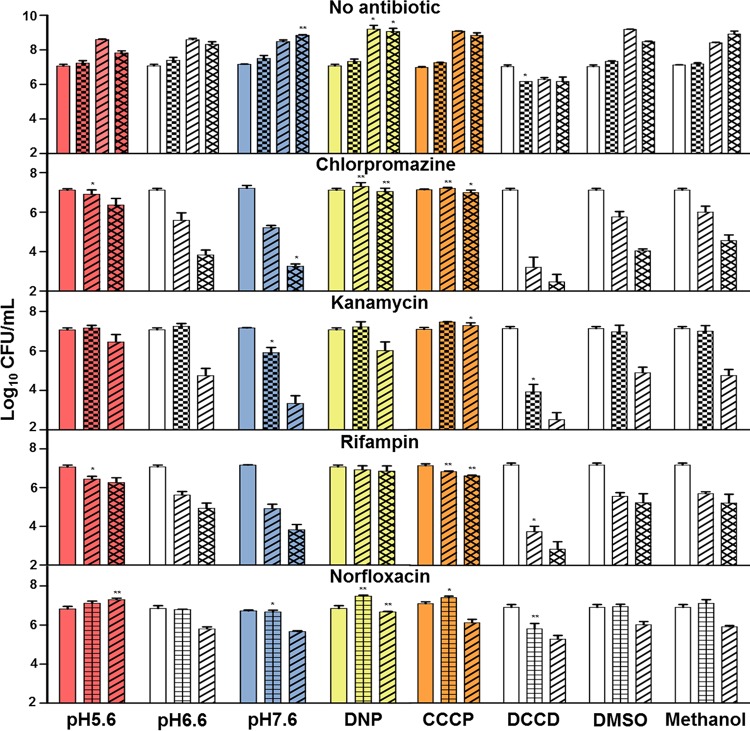
Conditions that countered intracellular alkalization increased *M. smegmatis* antibiotic tolerance. *M. smegmatis* was challenged with antibiotics over a pH range or in combination with DNP, CCCP, or DCCD at pH 6.6. Antibiotics were assayed in the presence of DMSO or methanol to control for vehicle effects. Antibiotics were administered at the concentrations listed in Table 3, and CFU were determined at 0 h (no pattern), 3 h (checkered), 12 h (gridded), 24 h (diagonal up), and 48 h (latticed). The data shown are means ± standard deviations from at least 3 replicates. All conditions were compared to the respective pH 6.6 time point for statistical *t* test analysis (*, *P* < 0.05; **, *P* < 0.01).

The concept of proton homeostasis as an important aspect of antibiotic killing grew from our investigation of chlorpromazine. Chlorpromazine is a phenothiazine that is bactericidal against mycobacteria and has been shown to inhibit the essential respiratory enzyme Ndh, a type II NADH dehydrogenase (Ndh2) (16). The functional difference between Ndh2 and Ndh1 resides in the fact that Ndh1 is energy conserving and pumps four protons, while Ndh2 is unable to translocate protons (17). Thus, we predicted inhibition of Ndh2 should disrupt proton homeostasis by shifting NADH oxidation toward proton-translocating alternatives to Ndh2. To determine if the activity of chlorpromazine was dependent on excess proton translocation, a subinhibitory dose of the protonophore 2,4-dinitrophenol (DNP) was added to facilitate proton equalization across the membrane gradient. DNP levels were first titrated against *M. smegmatis* to determine a concentration that did not inhibit growth (Fig. 1). Use of a subinhibitory DNP dose ensured the effect of the protonophore was not a result of pH gradient dissipation and growth arrest. The bactericidal activity of chlorpromazine was entirely abrogated by protonophore addition but not by addition of vehicle, dimethyl sulfoxide (DMSO) (Fig. 1). A subinhibitory dose of a second protonophore, carbonyl cyanide *m*-chlorophenyl hydrazine (CCCP), also blocked antibiotic killing in a similar manner to DNP (Fig. 1). These findings indicate that the lethal effect of chlorpromazine could be from a hyperpolarized pH gradient and back-pressure on the ETS, or from a depletion of internal protons.

To distinguish between potential chlorpromazine mechanisms, killing was assayed over a range of extracellular pHs (pH_EX_). We found an alkaline extracellular environment enhanced chlorpromazine killing, while an acidic environment blocked lethality (Fig. 1). Thus, increasing extracellular protons prevented chlorpromazine killing, thereby demonstrating a hyperpolarized membrane was not the basis for cell death. The increased lethality by alkaline pressure on the bacilli suggested death from chlorpromazine treatment stemmed from depletion of cytoplasmic protons. As pH_EX_ influences pH_IN_ (18, 19), high pH_EX_ should exacerbate antibiotic-induced internal alkalization. Furthermore, changes in pH_EX_ and DNP had no effect on the MIC of chlorpromazine (Table 1). Therefore, increasing proton influx decreased chlorpromazine killing but did not reduce its growth inhibitory activity, demonstrating the antibiotic was active in the presence of the protonophore.

**TABLE 1:** Antibiotic MIC values in this study

Culture	MIC mode (µg/ml)^a^								
	Carb	Cpz	Gent^b^	Kan^b^	Levo	Nor	Rif	Tet	Vanc
*M. smegmatis*									
pH 5.6		50		2.0		4.0	6.3		
pH 6.6		50		2.0		2.0	25		
pH 7.6		50		1.0		2.0	25		
pH 6.6 + DNP		50		2.0		2.0	13		
*S. aureus*									
pH 6				50		3.1			13
pH 7				13		1.6			13
pH 8				3.1		1.6			13
pH 7 + DNP				200		0.4			13
*E. coli*									
pH 6	6.3			13		125			
pH 7	6.3			6.3		63			
pH 8	6.3			1.6		31			
pH 7 + DNP	13			6.3		125			
*P. aeruginosa*									
pH 6			3.1		0.2			13	
pH 7			1.6		0.2			13	
pH 8			0.4		0.1			13	
pH 7 + DNP			1.6		0.2			13	

a Carb, carbenicillin; Cpz, chlorpromazine; Gent, gentamicin; Kan, kanamycin; Levo, levofloxacin; Nor, norfloxacin; Rif, rifampin; Tet, tetracycline; Vanc, vancomyin.

b MIC values for aminoglycoside antibiotics were higher under conditions that lowered PMF and therefore drug uptake (low pH_EX_ and DNP addition), which led to increased drug tolerance.

### Manipulation of pH homeostasis blocked lethality of several classes of antibiotics.

**FIG 2:**
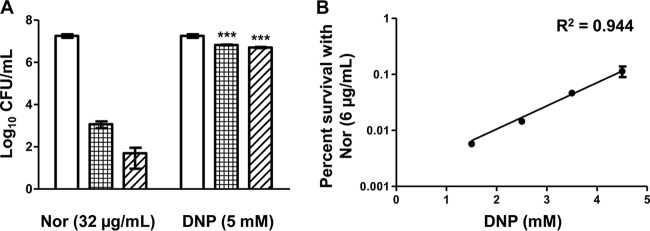
The protonophore concentration required for rescue was proportional to the antibiotic concentration. (A) *M. smegmatis* cultures were grown in the presence of 32 µg/ml norfloxacin (4× the calculated MBC) both with and without 5 mM DNP. CFU were determined at 0 h (no pattern), 12 h (gridded), and 24 h (diagonal up). (B) A second set of cultures were exposed to 6 µg/ml norfloxacin across a range of DNP concentrations, and CFU were determined after 24 h of exposure. For statistical analysis on panel A, a *t* test was used. For statistical analysis on panel B, a Pearson’s correlation coefficient was determined (***, *P* < 0.001).

Antibiotics that target *M. smegmatis* DNA, RNA, and protein synthesis were assayed to explore a role in pH homeostasis for drugs that do not directly target proton translocation pathways (Fig. 1). Surprisingly, the lethality of these antibiotics was also blocked with addition of DNP, and all antibiotics demonstrated reduced killing at lower pH_EX_, while displaying greater killing at higher pH_EX_. When over 10-fold additional norfloxacin was added to *M. smegmatis* culture, rescue was achieved through addition of an increased dose of DNP (Fig. 2A). The rescue of norfloxacin killing by DNP was directly proportional to the DNP concentration (Fig. 2B). These results suggest that the disruption of pH homeostasis may be a common attribute of antibiotic bactericidal activity in *M. smegmatis*.

### Manipulation of pH homeostasis blocked killing but not bacteriostatic activity.

To determine if changes in environmental pH or protonophore activity affected the overall mechanism of action of the antibiotics, we determined the MIC of each antibiotic under the conditions utilized for the killing assays (Table 1). An acidic pH_EX_ reduced antibiotic killing of *M. smegmatis* but had no effect on the MIC for chlorpromazine and kanamycin and decreased the MIC of rifampin from 25  µg/ml to 6.3 µg/ml. Only for norfloxacin did the MIC increase from 2 to 4 µg/ml with acidic pH_EX_. Addition of a protonophore, which blocked antibiotic killing, did not increase the MIC for any of the four antibiotics. These findings indicate the loss of bactericidal activity was not a result of the inability of antibiotics to inhibit their primary targets as they maintained the ability to stop growth.

**FIG 3:**
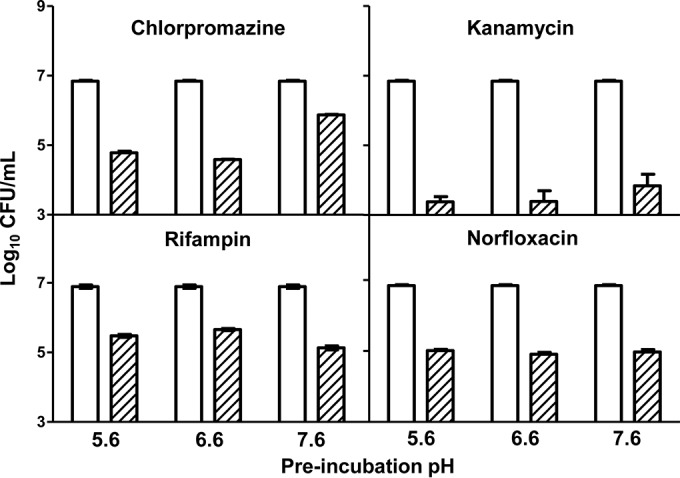
Loss of antibiotic lethality in acidic pH was not due to antibiotic degradation. Antibiotics were preincubated for 24 h at 37°C in DTA medium at pHs 5.6, 6.6, and 7.6. The pH was then adjusted to 6.6, and *M. smegmatis* was added to assay the drug efficacy post-drug incubation at each pH. The data shown are means ± standard deviations.

To determine if acidic conditions destabilized antibiotics, antibiotics were preincubated for 24 h at each pH, and killing assays were repeated after each pH was adjusted to the standard pH (Fig. 3). Preincubation of antibiotics at pH 5.6, 6.6, or 7.6 had little differential effect on antibiotic activity. Acidic conditions did not appreciably decrease antibiotic activity over the standard pH for any of the four antibiotics. Alkaline pH moderately decreased chlorpromazine and kanamycin activity, while rifampin was slightly more stable at alkaline pH compared to pHs 5.6 and 6.6. These findings are in agreement with the MIC data that demonstrated antibiotic bacteriostatic activity remained intact at low pH.

### Intracellular pH increased with antibiotics and decreased with protonophore.

**FIG 4:**
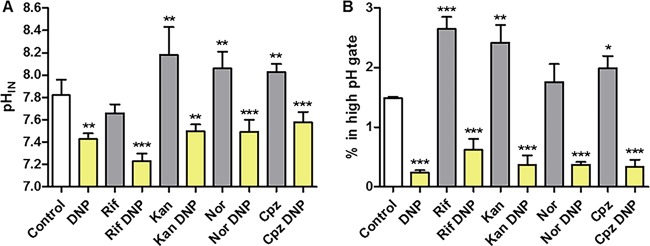
Single-cell flow cytometry analysis of pH_IN_. Cultures of *M. smegmatis* containing a ratiometric pH-sensitive GFP (20) were exposed to antibiotics (Cpz, chlorpromazine; Kan, kanamycin; Rif, rifampin; Nor, norfloxacin) with or without DNP, and individual cells were analyzed by flow cytometry. (A) The average pH_IN_ of the entire *M. smegmatis* population was determined as described previously (46). (B) Number of cells in the high-pH-gated population (pH of >8.6 [gate 1]) after cells were counted based on ratiometric fluorescence intensities. Samples were compared to the untreated control for statistical *t* test analysis (*, *P* < 0.05; **, *P* < 0.01; ***, *P* < 0.001).

*M. smegmatis* pH_IN_ was measured directly by flow cytometry using a pH-sensitive green fluorescent protein (GFP) (20). Direct pH_IN_ measurements demonstrated that 1.0 mM DNP had only a modest effect on pH_IN_. Even addition of 4-fold additional DNP failed to fully dissipate the pH gradient (see Fig. 11B and C). In contrast, the protonophore CCCP was highly effective at dissipating the pH gradient and was used to calibrate the GFP ratiometric signal (see Fig. 11A). pH_IN_ of the overall population rose from 7.7 to 8.1 and 8.2 for kanamycin, norfloxacin, and chlorpromazine, but not rifampin (Fig. 4A).

Unlike other drugs used against *M. smegmatis*, rifampin was unique in having an extremely high MBC/MIC ratio. While the MIC for rifampin was 25 µg/ml (Table 1), the MBC (minimum bactericidal concentration necessary to decrease CFU by 99.9%) was never observed even at concentrations as high as 1,600 µg/ml. Due to this extreme MBC/MIC ratio, rifampin is considered bacteriostatic against *M. smegmatis* (as defined by an MBC/MIC ratio of >4) (Table 1; Fig. 1) (21). In order to examine the killing activity of rifampin, a high concentration was used in this study (800 µg/ml) to achieve lethal levels (~99% decrease in CFU after 48 h).

Single-cell flow cytometry analysis demonstrated that 3 h of antibiotic exposure alkalized a subpopulation of bacilli (Fig. 4B). Examination of the subpopulation with a high pH_IN_ (≥8.6) demonstrated that all antibiotics, including rifampin, increased the number of bacilli within this high-pH_IN_ population. Addition of protonophore mitigated the alkalization caused by all antibiotics (Fig. 4). These findings suggest that upon antibiotic challenge, the bulk of the population resisted internal alkalization. However, over time, mechanisms used to resist alkalization failed, resulting in an unsustainable increase in pH_IN_ and subsequent cell death in this affected subpopulation. A large uniform increase in pH_IN_ was not observed—perhaps because a population-wide rise would likely result in a narrow temporal window of drug killing instead of the progressive bacterial death observed during antibiotic exposure.

### Proportion of cells in the high-pH populations correlated with antibiotic killing kinetics.

**FIG 5:**
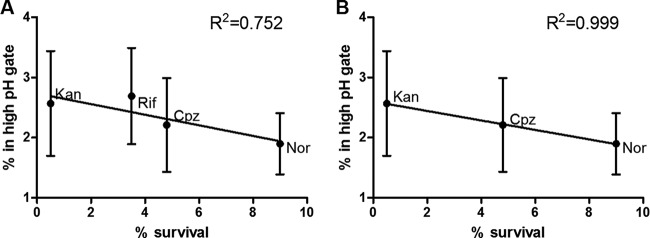
The rate of antibiotic killing was directly proportional to the efficiency of intracellular alkalization. (A) The percentage of survival was calculated for each antibiotic used with *M. smegmatis* (Fig. 1) at the 24-h time point. This was plotted against the proportion of cells observed in the high-pH population (Fig. 2), and a trend line was calculated with a Pearson’s correlation coefficient of *R*^2^ = 0.752. Kan, kanamycin; Rif, rifampin; Cpz, chlorpromazine; Nor, norfloxacin. (B) The same analysis was performed excluding rifampin, and a trend line was calculated with a Pearson’s correlation coefficient of *R*^2^ = 0.999.

Only a small proportion of cells displayed a high pH_IN_ after 1 h of antibiotic exposure (Fig. 4B), which would be expected as only a small percentage of bacilli neared death at 1 h. As each antibiotic caused a different proportion of cells to alkalize internally, and each antibiotic displayed different killing kinetics, we plotted the percentage of survival of each antibiotic after 24 h of exposure to the percentage of cells observed within the high-pH population. A correlation between the percentage of killing and the percentage of cells in the high-pH-gated population was observed (*R*^2^ = 0.752) (Fig. 5A). Rifampin was then removed from analysis due to the role of rifampin efflux pumps in *M. smegmatis* (22 – 24), which shift rifampin to a bacteriostatic antibiotic except at very high concentration. In the absence of the rifampin data, the correlation coefficient increased (*R*^2^ = 0.999) (Fig. 5B). The trend indicates that antibiotics that were more efficient at intracellular alkalization also demonstrated faster killing kinetics.

### Intracellular alkalization results in cell death.

**FIG 6:**
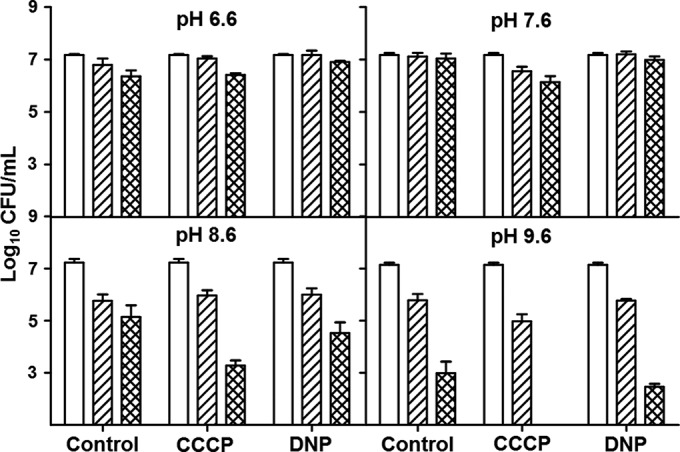
Alkalization alone caused cell death. *M. smegmatis* was exposed to an pH_EX_ of 6.6, 7.6, 8.6, or 9.6 with or without the protonophores DNP and CCCP, and samples were plated at 0 h (no pattern), 24 h (diagonal up), and 48 h (latticed). A *t* test was used for statistical analyses, and all conditions were compared to the same time point at pH 7.6 (approximate pH_IN_).

To investigate whether intracellular alkalization alone could cause death, *M. smegmatis* was exposed to phosphate citrate buffer at a pH near the pH_IN_, at 1 and 2 U higher or 1 U lower than the pH_IN_, and in the presence of the protonophores DNP and CCCP. Exposure of cells to a pH 1 U higher than the pH_IN_ led to an ~10-fold greater drop in viability than exposure to a pH_EX_ 1 U lower than the pH_IN_ (Fig. 6). Additionally, lethality was increased in the presence of either DNP or CCCP at pHs 8.6 and 9.6, but not at pH 7.6 or 6.6, further suggesting that intracellular alkalization could contribute to bacterial death observed during antibiotic treatment.

### Antibiotic killing was countered through acidification pressure on the pH_IN_ of diverse bacterial pathogens.

**FIG 7:**
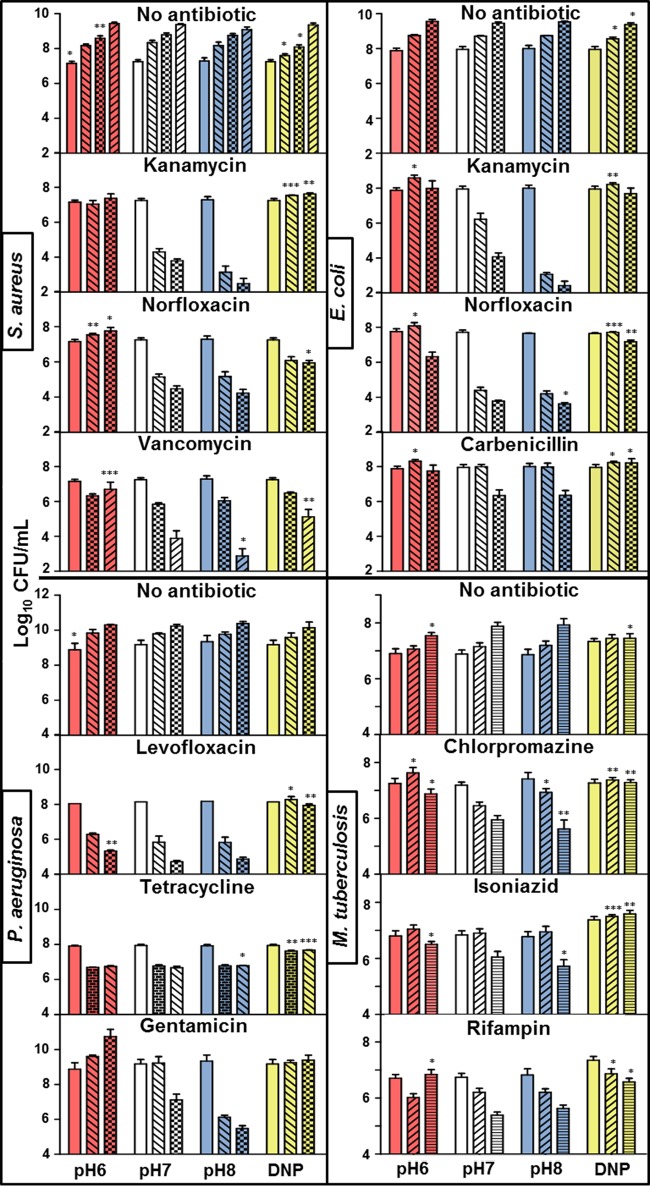
Shifting pH homeostasis toward intracellular acidification inhibited antibiotic killing of Gram-negative, Gram-positive, and acid-fast pathogens. *S. aureus*, *E. coli*, *P. aeruginosa*, and *M. tuberculosis* were challenged with antibiotics at concentrations listed in Table 3 over a pH range or at the mid-pH in combination with DNP. CFU were determined at 0 h (no pattern), 30 min (brick), 1.5 h (diagonal down), 3 h (checkered), 12 h (gridded), 24 h (diagonal up), and 72 h (light horizontal). A *t* test was used for statistical analysis, and all conditions were compared to the same time point at the standard mid-pH (*, *P* < 0.05; **, *P* < 0.01; ***, *P* < 0.001).

To test whether the observed pH homeostatic phenomenon was specifically a characteristic of *M. smegmatis* or potentially a more general characteristic of bactericidal antibiotics, *Pseudomonas aeruginosa*, *Escherichia coli*, *Staphylococcus aureus*, and *Mycobacterium tuberculosis* were subjected to drug treatments similar to those used for *M. smegmatis*. Antibiotics and antibiotic concentrations for *E. coli* and *S. aureus* were chosen based on a previously published study (3), and concentrations were determined for *P. aeruginosa* and *M. tuberculosis* that killed moderately at the neutral pH (~100 to 1,000-fold drop in CFU). A DNP concentration was determined for each species that did not affect growth and was used to ensure that growth arrest was not the basis of rescue (Fig. 7). As was the case for *M. smegmatis*, DNP inhibited killing from multiple antibiotics used against Gram-negative, Gram-positive, and acid-fast pathogens (Fig. 7). In most cases, the protonophore entirely abolished drug killing even though the drugs remained bacteriostatic (Fig. 7; Table 1). As found for *M. smegmatis*, a moderate decrease in pH_EX_ decreased antibiotic lethality, while a comparable increase in pH_EX_ increased lethality.

### Acidification pressure countered antibiotic killing under anoxic conditions.

**FIG 8:**
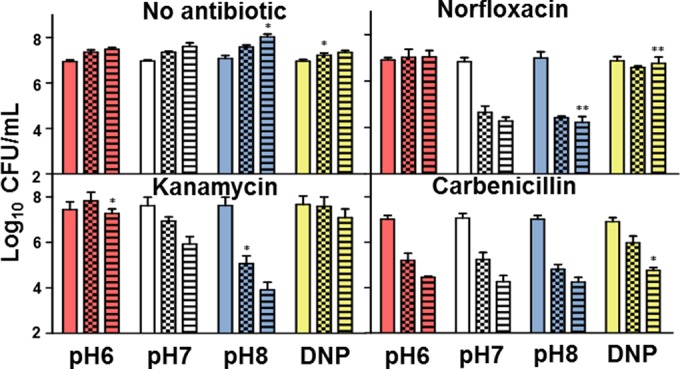
Anoxic antibiotic killing was inhibited by conditions that promoted intracellular acidification. Antibiotics (listed in Table 3) were added to anoxic cultures of *E. coli* in the presence or absence of DNP. CFU were determined at 0 h (no pattern), 3 h (checkered), or 6 h (heavy horizontal) for plating to determine survival by CFU. Kanamycin, an aminoglycoside, did not readily kill *E. coli* as a strong ΔΨ is required for aminoglycoside uptake (25). A *t* test was used for statistical analysis, and survival under each condition was compared to that at the same time point at pH 7 (*, *P* < 0.05; **, *P* < 0.01).

To ensure that the effect of pH homeostasis on antibiotic killing was not purely an aerobic phenomenon mediated via oxidative stress, anoxic, nitrate-respiring *E. coli* cultures were exposed to antibiotics in media of different pHs or in the presence of protonophore. As with aerobic cultures, both the acidic environment and the protonophore abrogated killing by antibiotics (Fig. 8). Nitrate addition ensured an active ETS; therefore, it is yet to be determined if pH homeostatic mechanisms could be involved in drug killing under fermentative conditions.

### Antibiotic uptake and bacteriostatic activity were not significantly affected by low pH_EX_ or protonophore activity.

**FIG 9:**
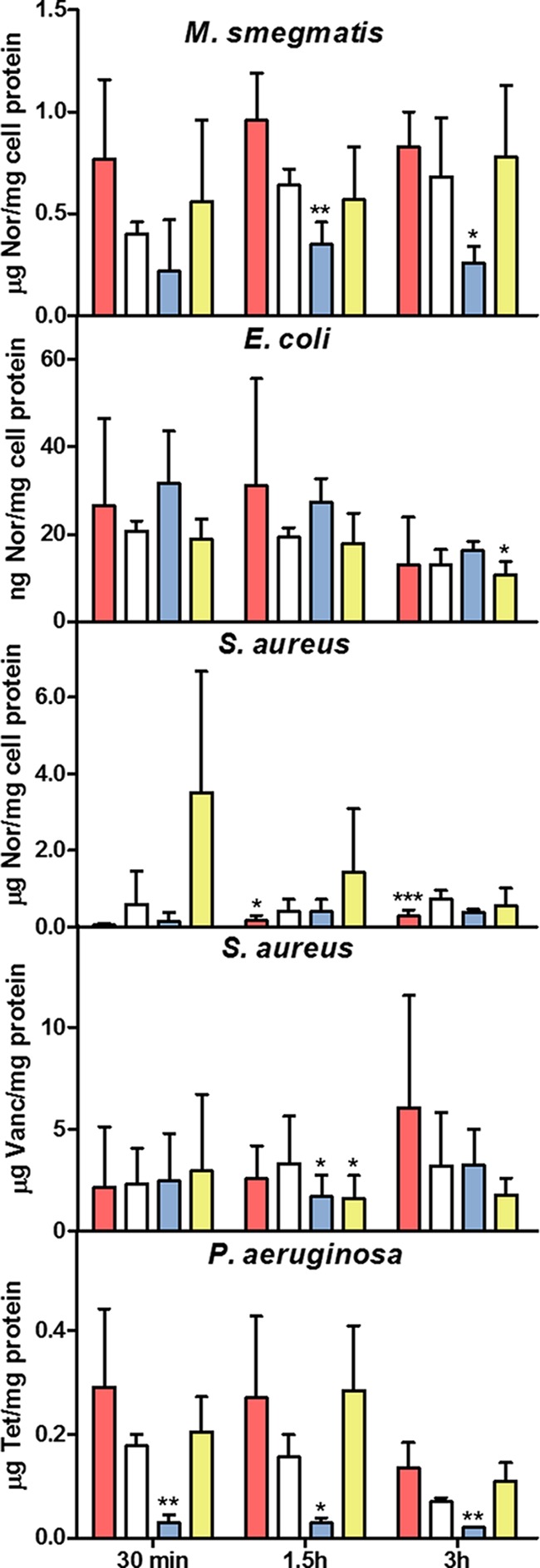
Antibiotic uptake was not decreased by acidic pH_EX_ or protonophore. [^3^H]norfloxacin (Nor) was added to the *M. smegmatis*, *E. coli*, or *S. aureus* culture, [^3^H]vancomycin (Vanc) was added to the *S. aureus* culture, and [^3^H]tetracycline (Tet) was added to the *P. aeruginosa* culture, and intracellular antibiotic concentrations were determined over time. The amount of added antibiotic was normalized to the intracellular protein concentration. For *E. coli*, *S. aureus*, and *P. aeruginosa*, pH 6 is in pink, pH 7 is in white, pH 8 is in blue, and pH 7 with DNP is in yellow. For *M. smegmatis*, pH 5.6 is in pink, pH 6.6 is in white, pH 7.6 is in blue, and pH 6.6 with DNP is in yellow. For statistical *t* test analyses, each condition was compared to the same time point at the mid-pH (*, *P* < 0.05; **, *P* < 0.01; ***, *P* < 0.001).

To test whether the observed phenomenon was simply an artifact of internal antibiotic concentration, ^3^H-labeled antibiotics were added to *E. coli*, *S. aureus*, *P. aeruginosa*, and *M. smegmatis* cultures in media with different pHs or in the presence of protonophore. Internal antibiotic concentrations were then calculated. We observed no statistical reduction in intracellular antibiotic levels either at acidic pH_EX_ or after addition of DNP, indicating lower drug uptake or retention was not likely responsible for the lack of antibiotic killing (Fig. 9). Uptake of kanamycin and gentamicin was not assayed as aminoglycoside antibiotics require a chemiosmotic gradient, or ΔΨ, which is a component of the total proton motive force (PMF [PMF = ΔΨ + ZpH]) for drug entry (25). Thus, the effect of DNP and low pH_EX_ on killing should have been at least partly due to reduced drug uptake for aminoglycoside antibiotics. A decrease in PMF by addition of DNP or acidic pH_EX_ should increase or have no effect on the intracellular concentration of most other drugs as a decrease in PMF reduces PMF-powered efflux pump activity necessary for drug removal from the cytosol (26 – 28).

To determine whether antibiotic activity was altered under low pH or in the presence of protonophore, MICs for all antibiotics were determined for *P. aeruginosa*, *E. coli*, *and S. aureus* under all conditions tested. As was observed in *M. smegmatis*, DNP and low pH_EX_ had little to no effect and sometimes lowered the MIC of antibiotics, indicating that the antibiotics were effective against their targets, even when bactericidal activity was blocked (Table 1).

### ATP levels increased with antibiotic treatment and decreased with inhibition of ATP synthase.

DNP rescues *E. coli* from killing by gyrase inhibitors, which was postulated to be due to the decrease in cellular ATP pool (29). We therefore determined whether ATP levels decreased in *M. smegmatis* when treated with antibiotics and DNP or the ATPase disruptor *N*,*N*′-dicyclohexyl carbodiimide (DCCD). ATP levels in antibiotic-treated *M. smegmatis* cultures were the same as or higher than those in the negative control (Table 2). ATP levels decreased slightly (less than 28%) when DNP was added in three of the antibiotic-treated cultures and increased with one antibiotic, reaching cellular ATP levels 2.4 times that of untreated bacteria. In addition, DCCD increased antibiotic bactericidal activity (Fig. 1), while it drastically reduced the levels of cellular ATP, indicating a decrease in ATP (Table 2) does not account for the loss of antibiotic killing.

**TABLE 2:** Measurement of intracellular ATP in *M. smegmatis*

Treatment^a^	Intracellular ATP concn^b^							
	Antibiotic only	Antibiotic with:						
		1 mM DNP	0.5 mM DCCD	0.5 mM DNP	2 mM DNP	4 mM DNP	50 µM CCCP	500 µM CCCP
Control	100 ± 0	64.0 ± 42.8	13.7 ± 3.2	85.5 ± 22.2	59.7 ± 36.2	48.7 ± 30.8	43.7 ± 9.4	41.8 ± 26.2
Cpz	102 ± 27	78.8 ± 22.5	7.70 ± 3.70					
Kan	102 ± 31	72.3 ± 15.7	13.1 ± 3.6					
Nor	122 ± 50	79.5 ± 17.0	15.1 ± 3.7					
Rif	233 ± 55	243 ± 82	24.7 ± 4.6					

a Cpz, chlorpromazine; Kan, kanamycin; Nor, norfloxacin; Rif, rifampin.

b One-hour samples were harvested and ATP measurements taken. Data are an average from six biological replicates read in duplicate ± standard deviation.

## DISCUSSION

**FIG 10:**
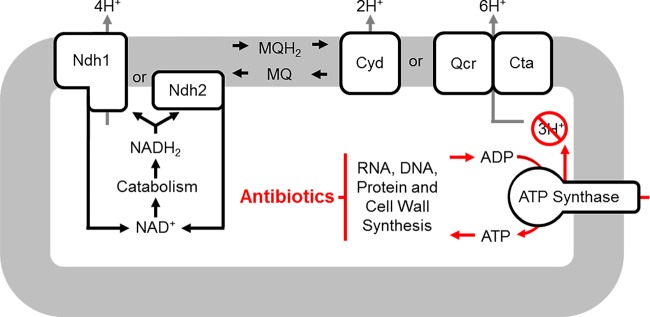
pH homeostatic hypothesis of antibiotic lethality. In the absence of antibiotics, nutrient-replete conditions sustain reduction of electron carriers and drive proton translocation via the ETS. Protons return to the cytosol through ATP synthesis. Bactericidal antibiotics target ATP-consuming reactions in a manner that fails to coordinately reduce the rate of proton translocation. Intracellular alkalization follows as the rate of ATP synthesis abates from an overall reduction in all biosynthetic reactions. Bacteria die as countermeasures to intracellular alkalization fail and pH_IN_ increases beyond viable limits.

Generation of an energized membrane by proton translocation is a primary output of energy metabolism and is required for many forms of life. We propose a model in which antibiotics kill bacteria, at least in part, through disruption of major cellular biosynthetic processes, which in turn drives an imbalance in proton homeostasis leading to lethal changes in pH_IN_ (Fig. 10). In this model, antibiotic disruption of DNA, RNA, protein, or cell wall biosynthesis subsequently decreases ATP consumption from all biosynthetic cellular processes due to an overall cessation of growth, resulting in a global reduction in ATP turnover. Indeed, bacterial cells exposed to bactericidal antibiotics display an increase in cytosolic ATP levels (30 – 32). We also confirmed that ATP levels increased upon antibiotic exposure. However, in our model the decrease in flux through the ATP synthase is of greater importance than the absolute level of ATP *per se*. In the absence of a concomitant reduction in respiration during the loss of ATP synthesis, protons would be drained from the cytosol and not replaced via ATP synthesis. The pH homeostatic hypothesis proposes that in order to determine if an antibiotic is subinhibitory, bacteriostatic, or bactericidal, all factors that affect pH_IN_ need to be considered. These include not only the overall inhibitory activity of the antibiotic on its target but also respiration rate, countermeasures employed to maintain pH homeostasis, the effects of drug influx and efflux on pH_IN_ and many other factors that influence the concentration of intracellular protons which may be specific to each bacterial species.

Herein we show that antibiotics directly caused an increase in *M. smegmatis* pH_IN_ and pushed a subset of the population to alkalize internally. The percentage of the population that alkalized after antibiotic stress was directly proportional to the rate at which each antibiotic killed. Conditions that promoted proton influx, such as a low pH_EX_ or the addition of a protonophore, caused antibiotic tolerance, whereas conditions that reduced proton influx, such as a high pH_EX_ caused an increase in antibiotic sensitivity. We also demonstrated that an increase in pH_IN_ was highly lethal, even in the absence of antibiotics.

The proton homeostatic hypothesis predicts that a key aspect of bactericidal antibiotics is a decrease in ATP flux in the absence of triggering a reduction in proton translocation. Bacteria have a variety of mechanisms to maintain proton homeostasis when facing alkalization pressure (33); however, if these mechanisms and proton reentry from ATP synthesis are outpaced by proton pumping, pH_IN_ will eventually alkalize to a point where hydrogen bonds between membrane lipids become disrupted or other essential processes collapse. Indeed, alkaliphiles synthesize high levels of cardiolipins, potentially to trap protons at the membrane surface in order to prevent membrane disruption in the face of severe environmental proton deficiency (33). In further support of the proton homeostatic hypothesis, antibiotic treatment increased ATP levels, and low doses of the ATP synthase inhibitor were synergistic with the killing activity of antibiotics. Additionally, we found that DNP was capable of inhibiting antibiotic killing under anoxic conditions, indicating the proton homeostatic hypothesis could account for antibiotic lethality in the absence of oxidative stress. However, additional experiments will be required to establish if pH homeostatic mechanisms influence bactericidal activity under fermentative conditions, during which the ATP synthase often runs in reverse, using ATP to pump protons across the bacterial inner membrane.

DNP was used at concentrations that did not affect cell growth rate to reduce the possibility that DNP induced tolerance due to growth arrest. Furthermore, a second protonophore, CCCP, also inhibited the bactericidal activity of antibiotics, indicating that specific pleiotropic effects of DNP were also not likely responsible for countering antibiotic killing. It could be argued that addition of DNP decreased intracellular ATP, which in turn decreased antibiotic target activity, thus affecting antibiotic killing. However, the DNP concentrations used in this study did not collapse the proton gradient, and DNP caused only a minor reduction in cellular ATP levels with three of the four antibiotics, and in one case, cellular ATP levels were more than double those of the untreated control. Furthermore, an additive effect was observed for all antibiotics in the presence of an ATPase inhibitor, which caused a far more dramatic drop in ATP than any protonophore-antibiotic combination. DCCD prevents ATP synthesis by blocking proton flow into the cell (9, 34, 35), which the proton homeostatic model predicts should exacerbate antibiotic killing by decreasing proton influx. Together, these results indicate that a decrease in ATP does not likely account for the rescue of antibiotic killing provided by DNP.

Flow cytometry demonstrated that all antibiotics tested, with the exception of rifampin, caused an overall average increase in pH_IN_ of the population. Although lethal levels of rifampin did increase the subpopulation of bacilli in an alkaline state, *M. smegmatis* is incredibly resistant to rifampin, with an MBC/MIC ratio over 84, well above the ratio in which antibiotics are considered bacteriostatic (21). Increased resistance of *M. smegmatis* to rifampin is due to LfrA and EfpA, members of the proton antiporter family (24, 36). The proton influx used to expel rifampin not only results in high rifampin resistance, but would be expected to contribute to a decrease in pH_IN_ from the influx of protons to drive the efflux pumps. As most antibiotic efflux pumps are either proton antiporters or hydrolyze ATP in order to function (22, 23), the effectiveness of these efflux pumps in preventing killing may be partially due to a role in decreasing pH_IN_. Although rifampin functions primarily as a static drug in *M. smegmatis*, a high dose was used to obtain lethality in this study to isolate the effects of pH on bactericidal antibiotic activity. ATP levels increased 2.3-fold after rifampin exposure, while exposure to the three bactericidal antibiotics modestly raised ATP levels (<20%). Since the level of rifampin was 84 times higher than the MIC necessary to stop growth, it is likely that all rifampin RNA polymerase targets were saturated, resulting in maximum decrease in ATP utilization. In addition, the expected population-wide increase in pH_IN_ from loss of ATP flux could have been largely offset from proton influx in exchange for rifampin efflux. However, the subpopulation increase with an alkaline pH_IN_ at lethal levels of rifampin indicated that a fraction of the population lost the ability to maintain pH homeostasis, resulting in death. These differences between rifampin and the three bactericidal antibiotics may indicate fundamental differences in bacteriostatic and bactericidal antibiotics. It was recently reported that bactericidal antibiotics accelerate respiration, while bacteriostatic drugs either suppress or have no effect on respiration (3, 37). It has been shown that the bacteriostatic antibiotic chloramphenicol targets the 50S ribosome subunit, but also decreases respiration by an unknown mechanism (38). The mechanisms involved in increasing or decreasing respiration by bactericidal versus bacteriostatic antibiotics are not well understood. However, the finding that the rate of respiration influences whether an antibiotic is lethal supports the importance of proton-translocating pathways in antibiotic killing.

As antibiotics kill a population slowly over time, rather than causing immediate sterilization, one would expect only a small proportion of the population at any point in time to display an alkaline cytosol. This is in fact what was observed when we measured pH_IN_ of individual cells. Additionally, it could be predicted that antibiotics with faster killing kinetics would cause a larger proportion of a population to alkalize. This phenomenon was observed with a strong correlation between the rate of bactericidal antibiotic killing and the proportion of cells with an alkaline cytosol.

It has been postulated that antibiotics have a common bactericidal mechanism that emanates from the production of oxidative stress generated from increased aerobic respiration (3, 11, 39). Kohanski et al. demonstrated that the antioxidant thiourea and the iron chelator 2,2′-dipyridyl blocked killing by antibiotics (3, 11). The dye hydroxyphenol fluorescein (HPF) was used as a hydroxyl radical intracellular probe that indicated an increase in oxidative stress during antibiotic killing (3). Two subsequent studies questioned the oxidative stress hypothesis, pointing to the fact that antibiotics are bactericidal in the absence of oxygen (40, 41). It was also demonstrated that thiourea protects anaerobic respiring bacteria from antibiotic killing (41). We propose that the ability of antioxidants like thiourea and iron chelators to suppress respiration (42, 43), and thus proton translocation, may be the basis of their protection against antibiotic killing rather than oxidative stress *per se*. In addition, HPF is also known to function as a pH sensor, and HPF demonstrated a shift under anaerobic conditions in absorbance after antibiotic challenge, consistent with an increase in pH_IN_ (41, 44), further supporting cytosol alkalization in antibiotic-treated cells. Hydrogen sulfide and nitric oxide have also been shown to rescue antibiotic lethality (4, 5). Importantly, these gases are powerful inhibitors of aerobic respiration via binding to cytochrome oxidase (15) and thus will dramatically reduce aerobic proton translocation. In addition, respiratory enzyme mutants confer antibiotic tolerance, especially those that result in a ratio shift in protons pumped per electron traversing the ETS (37, 39, 45). The chemistries of electrons and protons are closely linked, which makes it difficult to decipher if effects are due to oxidative stress or disruption of pH homeostasis. The pH homeostatic hypothesis is consistent with many of the experimental findings that suggest antibiotics kill via oxidative stress and with the compelling arguments that have questioned the role of oxidative stress as the common factor in the bactericidal activity. The oxidative stress and the pH homeostatic hypotheses of antibiotic killing are not mutually exclusive, and consideration of the effects of pH may explain some deficiencies in the oxidative stress model.

Our experimental evidence suggests a link between antibiotic bactericidal activity and pH homeostasis. However, only a limited number of antibiotics were investigated, and the most compelling experiments indicating antibiotic effects on pH_IN_ were conducted only in *M. smegmatis*. We also did not investigate the role of pH on antibiotic killing of strict anaerobes or under anaerobic conditions in which nonrespiratory pathways are used to generate a pH gradient. As perturbations of proton homeostasis have global effects on bacterial metabolism, alternative explanations will need to be further investigated to challenge or validate the role of protons in antibiotic killing. The pH homeostatic hypothesis provides a novel framework to view the bactericidal activity of antibiotics that, if correct, could be critical to the interpretation of numerous conditions that influence antibiotic lethality, and to understand mechanisms employed by persisters to tolerate a wide range of antibiotics.

## MATERIALS AND METHODS

### Culture conditions and strains.

Overnight cultures of *E. coli* MG1655, *S. aureus* ATCC 25923, and *P. aeruginosa* PAO1 grown in lysogeny broth Lennox (pH 7) (LB medium) were diluted 1:50 in LB medium at pH 6 (adjusted with hydrochloric acid), 7, or 8 (adjusted with sodium hydroxide) in 125-ml Erlenmeyer flasks. Cultures were allowed to grow while shaking for 1.5 h, and diluted to an optical density at 600 nm (OD_600_) of 0.1 with LB medium of the same pH. One-milliliter culture aliquots were added to 15-ml snap-cap tubes, antibiotics were added at concentrations listed in Table 3, and samples were incubated at 37°C shaking at 250 rpm. One hundred microliters of each experimental sample was collected at different time points via centrifugation at 16,000 × *g* for 1 min and suspended in LB medium, and survival was assayed by serial dilution and plating on LB agar plates. For *M. smegmatis* mc^2^155, overnight cultures grown in Dubos-Tween-albumin (DTA) medium (Difco Dubos broth base [Becton Dickinson], 0.5% bovine serum albumin fraction V, 0.75% glucose, 0.17% NaCl, 0.05% Tween 80) were diluted to an OD_600_ of 0.03 in DTA at pH 5.6 (adjusted with hydrochloric acid) or pH 6.6 or 7.6 (both adjusted with sodium hydroxide) and allowed to grow at 37°C in 125-ml Erlenmeyer flasks for 1.5 h while being stirred at 150 rpm. One-milliliter aliquots were added to 15-ml snap-cap tubes, and antibiotics and other drugs were added at the concentrations listed in Table 3. DCCD was added at a final concentration of 500 µM, and CCCP was added at a final concentration of 50 µM for the experiments illustrated in Fig. 1. Cultures were incubated with shaking at 250 rpm at 37°C. One hundred microliters of each experimental sample was collected at different time points via centrifugation at 16,000 × *g* for 1 min and suspended in DTA medium, and survival was assayed by serial dilution and plating onto DTA agar plates containing 4 g/liter of activated charcoal. Mid-log-phase (OD_600_ of 0.2 to 0.4) cultures of *M. tuberculosis* H37Rv grown in DTA medium were diluted in DTA to an OD_600_ of 0.05 at pH 5.6, 6.6, or 7, and 1-ml aliquots were added to sterile glass tubes (20 by 125 mm) containing stir bars (12 mm by 4.5 mm). Cultures were outgrown 1 h at 37°C, antibiotics were added, and samples were stirred rapidly (200 rpm) using a rotary magnetic tumble stirrer (V and P Scientific, San Diego, CA). Samples were taken at intervals, serially diluted, and plated on DTA agar plates containing 4 g/liter of activated charcoal to determine survival.

**TABLE 3:** Antibiotic concentrations used in this study

Antibiotic^a^	Concn in culture					
	*M. smegmatis*	*M. tuberculosis*	*S. aureus*	*P. aeruginosa*	*E. coli* aerobic	*E. coli* anoxic
Carb					10 µg/ml	40 µg/ml
Cpz	60 µg/ml	50 µg/ml				
DNP	1 mM	500 µM	100 µM	2 mM	250 µM	500 µM
Gent				2 µg/ml		
Kan	2 µg/ml		15 µg/ml		5 µg/ml	5 µg/ml
Inh	1 mg/ml	10 µg/ml				
Levo				0.7 µg/ml		
Nor	3 µg/ml		2 µg/ml		100 ng/ml	150 ng/ml
Rif	800 µg/ml	8 µg/ml				
Tet				50 µg/ml		
Vanc			7 µg/ml			

a Carb, carbenicillin; Cpz, chlorpromazine; DNP, dinitrophenol; Gent, gentamicin; Kan, kanamycin; Inh, isoniazid; Levo, levofloxacin; Nor, norfloxacin; Rif, rifampin; Tet, tetracycline; Vanc, vancomycin.

To determine the relative stability of antibiotics at pH 5.6, 6.6, and 7.6, each antibiotic was preincubated in DTA medium for 24 h at each pH. The media containing drugs at pHs 5.6 and 7.6 were then adjusted to pH 6.6 followed by filter sterilization. Pelleted *M. smegmatis* cells were suspended in preincubated DTA containing antibiotic, and the killing assays were conducted as described for the experiments shown in Fig. 1.

For anoxic growth, *E. coli* MG1655 single colonies were inoculated in 25-ml M9 minimal medium at pH 7 supplemented with 0.2% (wt/vol) succinate and 0.2% (wt/vol) d-lactate in 125-ml Erlenmeyer flasks and grown overnight at 37°C with shaking at 250 rpm. Cultures were diluted 1:50 into 10-ml anoxic M9 minimal medium supplemented with 0.2% (wt/vol) succinate, 0.2% (wt/vol) d-lactate, 400 mM sodium nitrate, and 0.1% (vol/vol) Oxyrase in 16-ml Hungate tubes with a tuberculin syringe through a butyl rubber septum stopper. Cultures were grown at 37°C for 16 h with shaking at 250 rpm, and diluted to an OD_600_ of 0.01 via the Hungate technique in fresh anoxic M9 medium at pH 6, 7, or 8 supplemented with 0.2% (wt/vol) succinate, 0.2% (wt/vol) d-lactate, 400 mM sodium nitrate and 0.1% (vol/vol) Oxyrase. Cultures were outgrown for 1.5 h, and antibiotics were added via syringe at concentrations listed in Table 3. Select cultures were supplemented with resazurin to ensure complete removal of oxygen. Samples were taken at different time points, and survival was assayed on LB agar plates.

To assay survival in phosphate citrate buffer of different pHs, overnight cultures of *M. smegmatis* were diluted to an OD_600_ of 0.05 and subjected to centrifugation, and pellets were suspended in phosphate-citrate buffer at pH 6.6, 7.6, 8.6, or 9.6. One-milliliter aliquots were added to 15-ml snap-cap tubes, and 25 µM CCCP or 1 mM DNP was added. Cultures were grown and survival determined as described above for *M. smegmatis*.

### MIC determination.

Overnight cultures were started for *E. coli*, *S. aureus*, *P. aeruginosa*, and *M. smegmatis* as described above. Bacteria were then exposed to 2-fold dilutions of antibiotics. Samples were plated at time zero and 3 h (for *E. coli*, *S. aureus*, and *P. aeruginosa*) or 24 h (for *M. smegmatis*). CFU were determined and recorded. The MIC was recorded as the minimum concentration at which the final CFU count was less than twice that at time zero. The values reported represent the mode of 3 biological replicates.

### Flow cytometry analysis.

**FIG 11:**
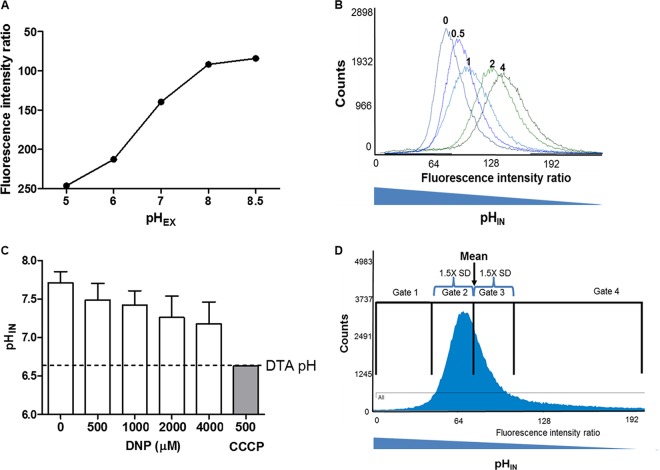
Flow cytometry analysis of DNP effect on proton gradient. (A) A sigmoidal standard curve was generated in the presence of CCCP similar to that observed previously (46). (B) DNP at 0, 0.5, 1, 2, or 4 mM DNP was added to *M. smegmatis* culture containing the ratiometric pH-sensitive GFP for 20 min, and cells were subsequently analyzed via flow cytometry. (C) pH_IN_ was calculated from the experiments shown in panel B. Even at the highest dose of 4,000 µM DNP (4-fold higher than the concentration used for *M. smegmatis* experiments), the pH_IN_ never reached the pH_EX_ of the medium. In contrast, 500 µM CCCP, a much stronger protonophore, equalized pH_IN_ to that of the medium within 20 min. (D) For flow cytometric gate placement, the mean fluorescence ratio of the untreated control and the standard deviation were calculated using Summit5.3 software. Gate 2 was set as the mean − 1.5 standard deviations from the mean. Gate 3 encompassed the mean + 1.5 standard deviations from the mean. Gate 1 included all cells with a lower fluorescence intensity ratio (higher pH_IN_) than gate 2, and gate 4 included all cells with a higher fluorescence intensity ratio (lower pH_IN_) than gate 3.

Liquid cultures of *M. smegmatis* containing a plasmid encoding the ratiometric pH-sensitive green fluorescent protein (GFP) described elsewhere (20, 46) were maintained in DTA medium containing hygromycin at 20 µg/ml. Overnight cultures were diluted in fresh DTA without hygromycin to an OD_600_ of 0.03 and were allowed to grow while being stirred at 150 rpm at 37°C for 1.5 h. Antibiotics with or without DNP were added to cultures at the concentrations listed in Table 3. Cultures were maintained shaking in a 37°C Gyrotory water bath shaker (New Brunswick Scientific) until used for flow cytometry analysis in order to maintain proper culture temperature and aeration. After 3 h of antibiotic exposure, flow cytometry was performed at the CU-Anschutz Medical Campus Cancer Center Flow Core on a Moflo XDP 70 cell sorter. Samples were maintained at 37°C throughout the analysis process. Data were collected on 1 × 10^5^ bacterium-size particles per sample. To place analysis gates, the mean fluorescence intensity ratio was obtained using the untreated control containing the ratiometric GFP. The standard deviation was calculated using Summit5.3 software, and 1.5 standard deviations from the mean in either direction were used to place the “normal” pH gates (gates 2 and 3 in Fig. 11D). Gate 1 (“high pH”) was defined as the gate with a lower fluorescence intensity ratio than gate 2; gate 4 included all cells with a higher fluorescence intensity ratio than gate 3. After all gates were set, cultures were counted, and Summit5.3 software was used to analyze samples. Number of events in the high-pH gate was divided by total counts to determine the proportion in the high-pH gate. Background fluorescence was determined by analyzing *M. smegmatis* lacking the ratiometric pH-sensitive GFP. For all data analysis, doublet cells were first excluded from samples via side scatter then analysis was restricted to GFP-positive cells. To calculate the pH_IN_ of all samples, a standard curve was generated as described elsewhere (Fig. 11A) (46). Antibiotics were added to samples as described above and analyzed via flow cytometry after 1 h of incubation, also as described. Internal pH values were determined by interpolating 410/470 fluorescence ratios on the standard curve. For average pH_IN_ measurements, each reported value represents the mean and standard deviation from 9 biological replicates for untreated and 8 biological replicates for all other conditions. Each reported high-pH population value represents the mean and standard deviation from 9 biological replicates for untreated, 5 biological replicates for treated with DNP, rifampin, rifampin plus DNP, and kanamycin, 4 biological replicates for treated with chlorpromazine, chlorpromazine plus DNP, and kanamycin plus DNP, and 3 biological replicates for treated with norfloxacin and norfloxacin plus DNP.

### Antibiotic uptake assays.

Antibiotic uptake assays were performed similarly to those previously described (47). Stock [^3^H]norfloxacin (31.3 mCi/mg [American Radiolabeled Chemicals]) was prepared by diluting labeled drug with unlabeled norfloxacin to specific activities of 9.8 mCi/mg for *E. coli* and 3.6 µCi/mg for *S. aureus*, and *M. smegmatis*. Working concentrations of 10 µg/ml for *E. coli* experiments and 100 µg/ml for *S. aureus* and *M. smegmatis* experiments were used. Stock [^3^H]vancomycin (0.133 mCi/mg [American Radiolabeled Chemicals]) was prepared by diluting labeled drug with unlabeled vancomycin to a specific activity of 0.011 mCi/mg and a working concentration of 1.0 mg/ml. Stock [^3^H]tetracycline (44 mCi/mg [American Radiolabeled Chemicals]) was prepared by diluting labeled drug with unlabeled tetracycline to a specific activity of 0.51 mCi/mg and a working concentration of 10 mg/ml. Cultures were started as described above*.* Stock solutions of [^3^H]norfloxacin, [^3^H]vancomycin, or [^3^H]tetracycline were added to achieve a final antibiotic concentration shown in Table 3. Aliquots of culture (450 µl) were removed at intervals, placed on GF/C filters (Whatman) while under vacuum filtration, and washed with 8 ml 0.85% NaCl. Filters were freeze-dried at −80°C. Radioactivity on filters was quantitated by scintillation counting. Three readings of all samples were recorded, and the average counts per minute was obtained. Nonspecific binding of ^3^H-labeled antibiotic to filters was determined by adding labeled antibiotic to cell-free medium and subjecting it to the same vacuum filtration and washing as experimental samples, which served as a negative control. Internalized antibiotic was calculated by subtracting the negative control from each sample. The counts from this sample were divided from the counts obtained by adding pure labeled antibiotic of a known concentration to a membrane without washing to obtain a concentration of antibiotic associated with cells. This antibiotic concentration was divided by the total cellular protein concentration for each sample to obtain a measurement of antibiotic per total protein.

To determine cellular protein concentration, non-radiolabeled norfloxacin, vancomycin, or tetracycline was added to cultures as described above, and aliquots were removed for protein content determination with a Bio-Rad protein assay kit (Bio-Rad Laboratories, Richmond, CA). Four hundred-fifty-microliter samples were taken at the same time points used for radioactive analysis and subjected to centrifugation at 16,000 × *g* for 1.5 min, and supernatants were carefully discarded. Pellets were suspended in equal volumes of 1× phosphate-buffered saline and subjected to lysis via sonication. Supernatants were clarified by centrifugation, and 150 µl was used for total protein analysis as per the manufacturer’s protocol (Bio-Rad QuickStart Bradford assay) in duplicate. The values from the duplicate samples were averaged per time point per biological replicate. Each reported value represents the mean ± standard deviation from 3 biological replicates for norfloxacin and tetracycline experiments and 6 biological replicates for vancomycin experiments.

### ATP quantification.

ATP measurements were taken as previously described (48). After 1 h of treatment, cultures were pelleted, frozen on dry ice, and stored at −80°C until processed. Frozen cell pellets were suspended in 100 µl 0.025 M HEPES buffer with 0.02% Tween 80 (pH 7.75) and added to glass tubes (12 by 75 mm) containing 40 µl chloroform. Mixtures were then heated at 80°C for 20 min, and subsequently, 4.9 ml 0.025 M HEPES buffer (pH 7.75) was added. The ATP assay was performed as per the manufacturer’s protocol (Promega Enliten ATP assay system). Ten-microliter samples were added to a 96-well plate (Costar solid white) and loaded into an L-Max microplate luminometer (Molecular Devices). Enliten luciferase-luciferin reagent dissolved in sample buffer was injected immediately before the results were read. Luminescence was determined every 0.1 s for a total of 100 s. Data are the averages from 6 independent experiments assayed in duplicate.

### Statistical analysis.

The Colorado Biostatistics Consortium at the Colorado School of Public Health was consulted to determine appropriate statistical analytical methods. For all statistical analyses, a paired, one-tailed *t* test was performed. For flow cytometry analyses, all data points were compared to the control sample from the same experiment. For survival studies, all data points were compared to the mid-pH sample from the same experiment unless otherwise noted.
